# The Shared Preference Niche of Sympatric Asiatic Black Bears and Sun Bears in a Tropical Forest Mosaic

**DOI:** 10.1371/journal.pone.0014509

**Published:** 2011-01-20

**Authors:** Robert Steinmetz, David L. Garshelis, Wanlop Chutipong, Naret Seuaturien

**Affiliations:** 1 Conservation Biology Unit, World Wide Fund for Nature Thailand, Bangkok, Thailand; 2 Minnesota Department of Natural Resources, Grand Rapids, Minnesota, United States of America; 3 Conservation Ecology Program, King Mongkut's University of Technology Thonburi, Bangkok, Thailand; University of Pretoria, South Africa

## Abstract

**Background:**

Ecologically similar species often coexist by partitioning use of habitats or resources. Such partitioning can occur through divergent or shared niches. We investigated overlap in habitat use and spatial co-occurrence by sympatric Asiatic black bears and sun bears in three habitats in Thailand, and thereby assessed which niche model best accounts for their coexistence.

**Methods/Principal Findings:**

We used density of species-specific signs to assess habitat use. Signs of both bear species occurred in all three habitats, and on >60% of sampling transects. Both species fed mostly on fruit; insect feeding signs were uncommon, and were mostly from sun bears. Significant differences in habitat use occurred only in montane forest, the habitat in which fruit was most abundant; incidence of black bear sign there was six times higher than that of sun bears. Habitat use was similar between the two species in the other habitats, which comprised 85% of the area. Of 10 habitat attributes examined, fruiting tree density was the best predictor of occurrence for both species. Models that included interspecific competition (fresh foraging activity of the other species) were less supported than the top models without competition.

**Conclusions/Significance:**

Bear species co-occurrence at both coarse and fine spatial scales and use of the same resources (fruit trees) indicated common niche preferences. However, their habitat use differed in ways expected from their physical differences: larger black bears dominated in the most fruit-rich habitat, and smaller sun bears used less-preferred insects. These results indicate broadly overlapping fundamental niches combined with asymmetric competition—features consistent with the concept of shared preference niches. This model of the niche has received little attention in ecology, but appears to be relatively common in nature.

## Introduction

Ecologically similar species may coexist by selecting different habitats or resources within the same landscape [1], [2]. One way such partitioning may be generated is through morphological or behavioral differences that predispose different species to be better adapted to certain habitats [3], [4], [5]. This model of coexistence, based on divergent niches and distinct preferences, is a common underlying assumption in community ecology [6]. Habitat partitioning can also arise when species share preferences for a resource or habitat, but differ in their tolerances or competitive abilities, so become differentially distributed along an environmental gradient [3]. Preference here refers to the portion of an environmental gradient where a species is most abundant, has highest fitness, or chooses to be [3], [4]. For example, pocket mice (*Perognathus longimembris*) and kangaroo rats (*Dipodomys merriami*) both prefer open desert where food is most abundant, but high densities of the larger-bodied kangaroo rats displace the mice to bush habitat with less food, where they can nonetheless successfully meet their energy requirements [7]. Many communities appear to be structured by this alternate model of the niche, based on shared preferences; coexistence is achieved not through divergent niches, but by a tradeoff between competitive dominance and the ability to survive and reproduce in habitats with lower concentrations of resources [4], [6]. Despite its prevalence in nature, the shared preference niche has received relatively little attention in ecology [3].

**Figure 1:**
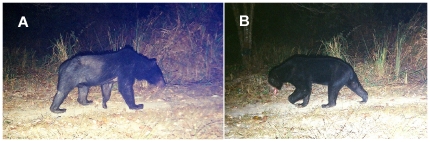
An Asiatic black bear (A) and sun bear (B) photographed at the same location in Thailand nine days apart (2009). These sympatric bear species co-occur at fine spatial scales, as seen here, throughout mainland Southeast Asia. Photographs: R. Steinmetz.

Asiatic black bears (*Ursus thibetanus*; hereafter, black bear) and sun bears (*Helarctos malayanus*) have coexisted in mainland Southeast Asia since the Middle Pleistocene [8], [9] and presently co-occur in northeast India, Myanmar, Thailand, Laos, Cambodia, Vietnam, and perhaps Bangladesh and southern China [10]. Within this region they co-occur at fine spatial scales (Fig. 1) such as within forest blocks as small as 80 km^2^ [11], [12]. Sun bears, at 40–60 kg, are about half the size of black bears (65–150 kg) [13], but the two species are ecologically and behaviorally similar. Both species are opportunistic omnivores that share broadly similar diets of insects and fruit, which they obtain from the ground and by climbing trees [14]–[16]. Given their ecological similarities and long history of shared ranges, how do these two species coexist?

In North America, sympatric brown bears (*U. arctos*) and American black bears (*U. americanus*) exhibit morphological and behavioral differences that render each species relatively more successful in different habitats [17], [18]. As a result, they coexist principally through differences in habitat use: brown bears often use open habitats, whereas American black bears prefer forests [19]–[21]. Thus, the distribution and relative abundance of these bear species is shaped by divergent niches that promote distinct preferences. In contrast, Asiatic black bears and sun bears are much more similar in most traits: both have curved claws for climbing, neither seems markedly more aggressive than the other, and both are forest-dwelling species. Habitat partitioning between them thus might occur through shared preferences, perhaps involving body-size mediated tradeoffs among different forest types or along gradients of resource abundance across forest types.

Using bear signs distinguished to species, we sampled the occurrence of sympatric black and sun bears in a mosaic of deciduous and evergreen habitats in Thailand. These species leave abundant sign in the forest, which is conspicuous, long-lasting, and related mostly to feeding. Signs most commonly encountered are claw marks on trees climbed for fruit, rest, or refuge, and diggings, opened termite mounds, and logs torn apart while foraging on invertebrates. Such signs result from behavioral decisions related to feeding or security, and thus are a good currency for quantifying habitat use and selection because they are linked directly to individual fitness. Bear signs are discrete ‘event sites’—places where animals have invested time and energy to accomplish important life functions [22]. McGill et al. [6] argued that community ecology should explore species interactions in terms of performance currencies that are linked to individual fitness. The foraging effort expended by bears, which is captured in bear sign, is such a measure.

We sought to compare the ecological niches of each species and to assess evidence for their coexistence through either niche differentiation or shared preferences. We compared niches by modeling habitat use of each species independently in the same area and comparing results [23]. If the two species of bears coexisted through divergent niches, we predicted that species-specific sign would (i) be associated with different forest types or (ii) correspond with different ecological attributes, resulting in different statistical models of habitat selection. Conversely, if sun and black bears had shared niches, we expected (i) widely overlapping use of habitats and resources, but also (ii) evidence of differential use of some resources related to the size differences of these two species. We analyzed habitat selection at two spatial scales. At a fine scale, bears select feeding or resting sites within habitat patches. At a coarser scale, bears choose among patches across a landscape mosaic. We related occurrence of bears to habitat variables measured at these two scales: in proximity to event sites, and at a scale corresponding to home range sizes of each species.

We also investigated interspecific relationships between these bears. Measuring the effects of competition on patterns of species coexistence requires manipulative experiments [1] that are impossible for rare species such as these. As an alternative, we assessed the importance of competition relative to habitat attributes by comparing models of habitat selection with and without foraging activity of the other species as a surrogate for potential interspecific competition. Best supported models should include activity of the other species if competitive interactions strongly influenced habitat selection. We further assessed interspecific relationships by examining co-occurrence of bear signs at a fine spatial scale [21]; spatial segregation of species-specific signs would suggest that the distribution of one or both species was constrained by the other. A supplementary objective was to describe habitat use by each species. Both species are threatened with extinction (Vulnerable [10]), and this was the first ecological study of each in mainland Southeast Asia, so basic information on habitat use may inform conservation decisions.

## Methods

### Study site

**Figure 2:**
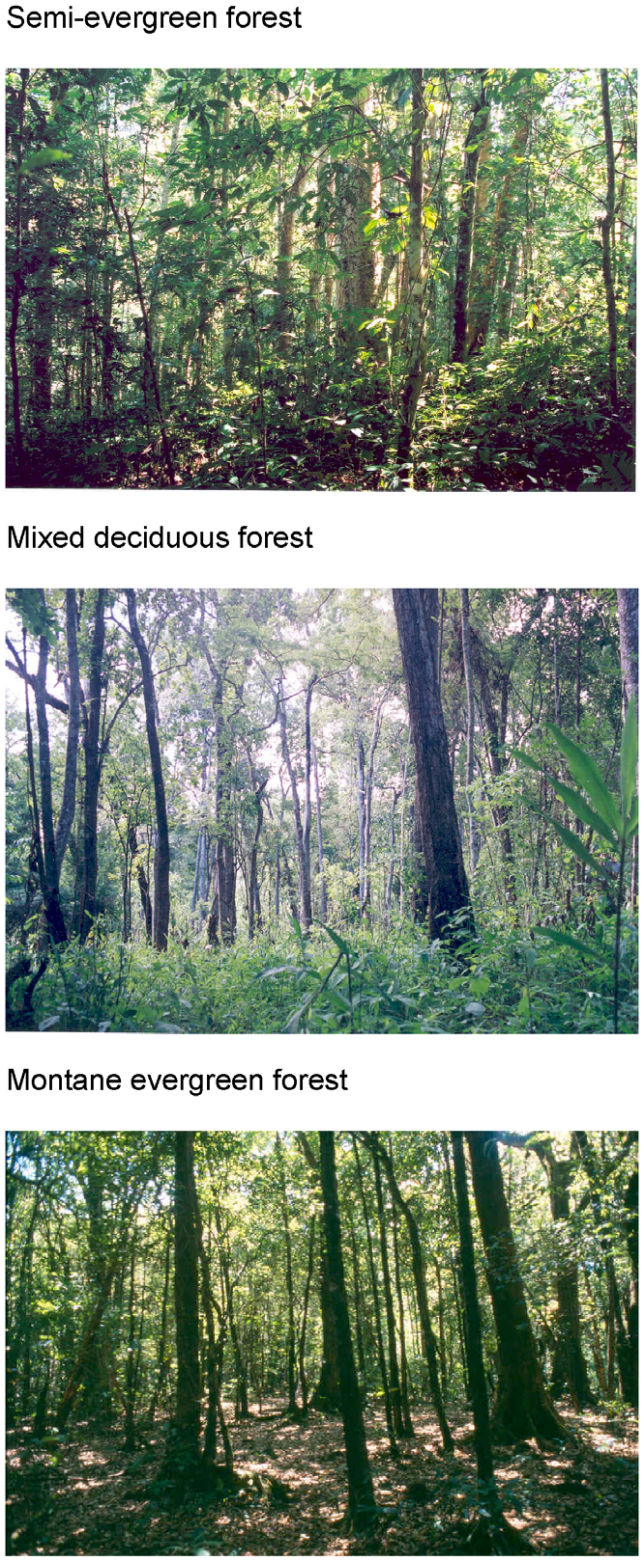
Forest types used by Asiatic black bears and sun bears in Thung Yai Naresuan Wildlife Sanctuary, Thailand, 2001–2003. (A) semi-evergreen forest, (B) mixed deciduous forest, (C) montane evergreen forest.

The 3,622 km^2^ Thung Yai Naresuan Wildlife Sanctuary (15° 00′–15° 23′ N, 98° 30′–99° 05′ E) is in western Thailand adjacent to Myanmar. The sanctuary is mountainous, with elevations up to 1811 m. Predominant forest types (Fig. 2) are mixed deciduous (54%), semi-evergreen (31%), and montane evergreen forest (15%) [24]. There are 3 seasons: cool and dry (November to February), hot and dry (March to May), and rainy (June to October). Mean annual rainfall during the study was 1731±217 mm (Thai Department of Meteorology, 2005), most falling between June and October. Mean annual maximum and minimum temperatures were 34°C and 21°C, respectively.

Semi-evergreen forest (SEF) and mixed deciduous forest (MDF) occur in a mosaic between 400 and 1000 m elevation. Tree density in SEF is almost twice that of MDF in Thung Yai [25]. SEF is tall, with densely-spaced broadleaf evergreen tree species that form a closed canopy at 25–40 m [26]. MDF is dominated by deciduous tree species, and canopy height can reach 30 m. Montane evergreen forest (MEF) occurs above 1000 m. It has high tree density like the SEF but is floristically and structurally distinct. MEF has a closed, even canopy of lower stature than SEF, and oaks (Fagaceae), an important bear food, are especially abundant. These forest types represent a gradient of food availability for bears. Fruit availability (density of trees bearing fruit) is highest in MEF, moderate in SEF, and lowest in MDF (see Results). Density of termite mounds, another potential bear food source, grades in the opposite direction: highest in MDF (5.3±10.3/ha), lower in SEF (1.2±1.7/ha), and near zero in MEF [27].

Four study sites, 15–30 km apart, were sampled. Three were at 500–900 m elevation and contained mosaics of SEF and MDF; the fourth, at 1200–1800 m, contained MEF. Transects to quantify bear sign were distributed over an area of 30–100 km^2^ at each site. The SEF and MDF sites were sampled 4 times between November 2001 and June 2003, spanning each of three seasons; data from the different seasons were pooled for analysis. MEF was sampled only in the hot dry season (March 2003). We established multiple sites to maximize the number of individual bears that would be included in our population-level data. Annual home ranges of adult sun bears and black bears are 6–21 km^2^ [28] and 30–150 km^2^ [29]–[31], respectively, and individual home ranges overlap widely. Thus, we expected the size and distribution of our study sites to encompass the activities of many individuals of each species.

### Observations of bear sign

We searched for bear signs in straight, 300-m long strip transects within homogeneous patches of each forest type. We conducted 38 transects in SEF, 27 in MDF, and 6 in MEF. To ensure good coverage of the forest mosaic at each site, we spaced transects at least 200 m apart. Transects covered the range of topographical variation within a study site (e.g., ridges, valleys). Although three of the study sites were sampled multiple times, no individual transects were sampled more than once.

Transects were 10 m wide in SEF and MEF (0.3 ha) and 20 m wide in MDF (0.6 ha), commensurate with differing tree densities. In each strip, we closely examined the trunk of every tree (>10 cm DBH), looking for bear claw marks, and also searched the ground for dug holes, opened termite mounds, or broken logs caused by bears foraging for insects.

### Distinguishing bear species and aging sign

Claw marks on trees climbed by black bears tend to be more widely spaced than claw marks of sun bears [32]. We measured the spacing of claw marks to classify whether the climbing event was by a black bear or sun bear, following the method in [32]. Small black bears and large sun bears make similar-sized claw marks; marks in this size range were categorized as indeterminant. This classification scheme was found to be 91–100% accurate when applied to bear-climbed trees in the wild [32]. Bear marks that were old and stretched with tree growth, indistinct, or not perpendicular to the tree trunk, were not identified to species. Bear footprints at insect-feeding sites were considered to be from black bears if hind pad width was >10 cm and total length >17 cm, from sun bears if measurements were below these thresholds, and indeterminate if length and width matched different species (R. Steinmetz, unpublished data from captive animals).

Based on previous experimental work [33], we distinguished marks that were fresh (<3 months), within-year, or older, based on the degree of bark regrowth in gouges. Insect foraging signs that were accompanied by footprints were categorized as fresh (<1 month), because bear footprints do not persist longer than 1 month (pers. obs.); all other insect-feeding signs on the ground were regarded as old (>1 month). Hence, we could separately analyze sign that was very fresh versus older.

### Habitat variables

#### Local scale

In each transect we measured six local scale variables: density of fruiting trees, fruit abundance, canopy height, canopy cover, ground cover, and elevation. We counted the number of fruiting trees (trees bearing fruit during sampling) of species and genera eaten by bears in Thung Yai (which we learned from a concurrent study on food habits [27]). We rated fruit abundance of each tree on a 1–4 scale (sparse to abundant); values per transect could range from 0 (i.e., no fruiting trees) to *n* (fruiting trees) ×4 (i.e., all fruiting trees had abundant crops). Canopy height, canopy cover, and ground cover were measured in two circular, 20-m diameter plots at 100 and 300 m along each strip transect (*n* = 142 plots total); transect means were the mean of these two plots. We estimated canopy height to the nearest 5 m. Canopy cover, defined as percent of ground covered by the horizontal projection of tree crowns within the plot, was subjectively classified by a single observer as <25%, 26–50%, 51–75%, or >75% (class midpoints were used for data analysis). We visually judged how well combined understory cover at 1 m height would hide a bear 10 m away and assigned scores of 1 (very sparse) to 5 (very dense; i.e., a bear would be completely concealed) in four cardinal directions; we used of the mean of these 4 values to represent the plot. Elevation was measured at transect centers.

#### Landscape scale

Landscape scale variables represented the environmental conditions that surrounded transects. Using ARCMAP software and a GIS database for the wildlife sanctuary, we measured the distance from transect center to (1) streams, (2) habitat patch edge (an index of habitat heterogeneity), and (3) sources of potential human hunting pressure (villages, or seasonal roads, whichever was closer). We calculated habitat composition, defined as percent evergreen forest (SEF or MEF) relative to MDF, within circular buffers centered at transect mid-points, with radii of 1.4, 2.6, 3.1, and 6.9 km for sun bears (min, max home range size) and black bears (min, max), respectively.

### Data analysis

#### Habitat use overview

We used density of signs (signs/ha), with transects as sampling units, as our metric of habitat use. We examined patterns of species-specific habitat use using fresh and within-year signs. Within each bear species, differences in sign density among the three habitats were evaluated using Kruskal-Wallis tests. Differences in sign density between bear species within each habitat were tested with Mann-Whitney *U*-tests. We assessed whether habitat attributes selected by each species differed by computing means for each attribute on transects with fresh signs, and testing for differences between bear species with Mann-Whitney *U*-tests. We used the Dunn-Sidak procedure to adjust family-wise Type 1 error rates for relevant sets of multiple comparisons [34]: between-bear sign density (6 comparisons) and local-scale habitat attributes (6 comparisons in each of 3 habitats) were considered significantly different between species if *P*<0.0009, and landscape-scale attributes (7 comparisons in each habitat) were considered different if *P*<0.007.

#### Habitat selection models

We used logistic regression to evaluate variables that distinguished used (sign recorded) from unused (sign not recorded) transects for each species, and thus identify habitat components selected by each species. Habitat selection models were developed using only fresh signs. A suite of 7–9, ecologically plausible, candidate logistic regression models were defined for each bear species and ranked using Akaike's Information Criterion (AIC). Covariates were entered into models together. The following models were considered: (1) all local and landscape variables, (2) landscape-level variables, (3) local-scale variables, (4) individual variables that emerged as potentially influential, based on relative size of beta coefficients in the above models. Additionally, we assessed top models (lowest AIC scores) with and without (a) fresh sign density of the other bear species as a surrogate for interspecific competition, and (b) forest type as a categorical predictor (combining MEF and SEF as evergreen forest). Finally, we postulated that avoidance of black bears by smaller-bodied sun bears might depend on ground cover for concealment [35]; therefore, for sun bears only, we included an interaction term between black bear activity (fresh signs/ha) and ground cover. Models were assessed based on lowest AIC scores and strength of evidence reflected in model weights, *w_i_* [36].

We assessed fit of models to the data using Hosmer-Lemeshow Goodness-of-Fit tests and by examining standardized residuals. We used Cook's distance to isolate individual cases that exerted undue influence on a model [34]. Predictive power of models was assessed using classification success rates. We controlled for multicollinearity by checking tolerance scores of variables; where tolerance was <0.2, we considered bivariate relationships with Spearman rank correlation and removed variables of lesser ecological relevance. We used regression slopes (*β*) and log-odds ratios to interpret the strength and direction of individual predictors in best-fit models.

Plot sizes were twice as large in MDF as in SEF and MEF, possibly increasing the probability that MDF plots would encompass at least 1 sign relative to other habitats. We examined the effect of plot size on regression results by entering plot size and the interaction between plot size and fruiting tree density. Resulting models showed no effect on probability of detecting bear signs for black bear (*X* ^2^ = 3.09, *P* = 0.21) or sun bear (*X* ^2^ = 1.2, *P* = 0.55).

#### Interspecific relationships

We examined interspecific relationships in 3 ways. First, we tested whether occurrence of each species was affected by foraging activity of the other species via logistic regression models. Second, we tested for nonrandom patterns of co-occurrence between bear species using *C*-scores calculated in EcoSim 7.0 [37]. The *C*-score index is *C_ij_* =  (*r_i_*−*S*)(*r_j_*−*S*), where *r_i_* and *r_j_* are numbers of sites (transects) with species *i* and *j*, and *S* is the number of shared sites [38]. This index measures the tendency for species to not occur together. In a community structured by competition, observed *C*-scores should be larger than expected by chance [37]. Differences between observed and expected *C*-scores were assessed through Monte Carlo simulations that randomized the occurrence of each species among sites (5000 iterations), using EcoSim 7.0. If sun bears and black bears used sites (transects) independently of one another, *C*-scores should not differ significantly from random. We conducted separate tests for both fresh and within-year signs, to examine potential competition at different temporal scales. Third, we used Spearman's rank correlation to test whether the amount of fresh foraging activity (signs/ha) by each bear species on the same transect was inversely related. Means are reported ± standard deviation (SD).

## Results

### Habitat use overview

Our transects covered 31.2 ha, in which we examined ∼15,000 trees. We recorded 675 bear signs: 92.3% (*n* = 623) were climbed trees (not including trees climbed for bee nests) and 7.7% (*n* = 52) were insect feeding signs (including trees climbed for bee nests). Overall sign density, including marks of all ages from both species of bears, was about three times higher in evergreen (SEF and MEF) than in deciduous (MDF) forests (Table 1; Kruskal-Wallis *X* ^2^ = 43.8, df  = 2, *P*<0.0001).

**Table 1:** Signs of sun bears and black bears (species combined; all sign ages) recorded in sign transects (*n* = 71) in three forest types of Thung Yai Naresuan Wildlife Sanctuary, Thailand, 2001–2003.

Sign type	Semi-evergreen			Mixed deciduous			Montane evergreen		
	*N* (%)	Density (sign/ha)	SD	*N* (%)	Density (sign/ha)	SD	*N* (%)	Density (sign/ha)	SD
Climbed trees	404 (94)	31.9	13.5	151 (84)	10.1	6.0	68 (100)	37.8	6.2
Insect feeding	17 (4)	1.6	3.4	10 (6)	0.6	1.2	0	0	0
Stingless bees	8 (2)	0.7	1.7	17 (10)	1.1	1.8	0	0	0
Signs combined	429 (100)	34.2	13.7	178 (100)	11.8	6.0	68 (100)	37.8	6.2

Insect feeding includes logs torn open, termite nests opened, and holes dug for terrestrial insects. Stingless bees refers to excavated nests of *Trigona* sp.

Claw marks on climbed trees were the predominant signs in each habitat, comprising 84% (MDF) to 100% (MEF) of our observations and occurring on 92% of all transects (Table 1). Insect-feeding signs were also widespread, occurring on about half of transects in SEF (52%) and MDF (42%). Raided stingless-bee (*Trigona* sp.) nests were 1.6 times denser in MDF than SEF (*U* = 416, *P* = 0.06), whereas terrestrial insect-feeding signs (dead wood opened, nests of ants and termites excavated) were denser, though highly clumped, in SEF (*U* = 503.5, *P* = 0.7; Table 1). Insect feeding signs were not detected in MEF. We collected 33 bear scats opportunistically during the study (unidentifiable to species); 79% (*n* = 26) contained fruit (70% with only fruit); the remainder contained insects (mainly ants and beetles) or vegetation.

Of 648 climbed trees (including those climbed to prey on stingless bees), 297 (46%) had within-year claw marks that were sufficiently distinct and complete to measure for species classification: 160 were identified as sun bear, 129 as black bear, and 8 were indeterminant. Nineteen percent of claw marks were fresh (created within 3 months). Thirteen of 27 (48%) terrestrial insect-eating signs and 4 of 25 (16%) stingless bee feedings were fresh. Nineteen (37%) insect-feeding signs could be identified to bear species. This subsample of within-year sign that was differentiated to species was used to investigate species-specific habitat use.

#### Species-specific habitat use

**Figure 3:**
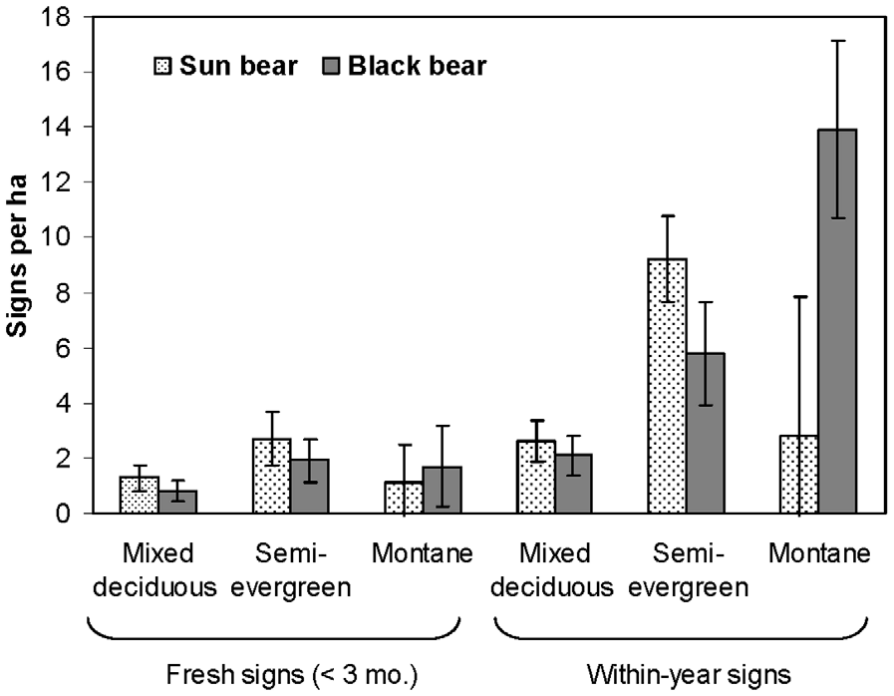
Sign density (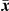 and 90% CIs) of sun bears and black bears in three habitats of Thung Yai Naresuan Wildlife Sanctuary, Thailand, 2001–2003. Fresh signs (<3 months) are a subset of within-year signs.

Fresh sun bear signs were over twice as abundant in SEF (2.7±3.5/ha) as in MDF (1.3±1.4) or MEF (1.1±1.7) (Fig. 3). Fresh black bear signs were about twice as abundant in SEF (1.9±2.9/ha) and MEF (1.7±1.8) as in MDF (0.8±1.1). These differences were not statistically significant for either sun bear (*X* ^2^ = 1.98, df  = 2, *P* = 0.37) or black bear (*X* ^2^ = 1.81, df  = 2, *P* = 0.41). However, considering sign up to a year old, foraging activity by black bears was significantly higher in MEF (13.9/ha) than in SEF or MDF (*X* ^2^ = 19.85, df  = 2, *P*<0.0001), whereas sun bear activity was highest in SEF (9.2/ha; *X* ^2^ = 27.23, df  = 2, *P*<0.0001; Fig. 3).

#### Interspecific differences in habitat use

Sun bears produced 19–37% more signs/ha than black bears in SEF and MDF (Fig. 3); differences were nearly significant for within-year SEF signs (*U* = 480.5, *P* = 0.01), but not for other comparisons (*P*>0.25). Conversely, black bears were predominant in MEF (Fig. 3): within-year black bear signs were six times more abundant than sun bears (*U* = 2.0, *P* = 0.009).

Most identifiable insect-feeding signs (16 of 19 = 84%), arboreal (stingless bees) and terrestrial (digging, log-opening) in both SEF and MDF, were from sun bears. Another 11 insect-feeding signs found off transects also were from sun bears.

Transects with fresh signs of sun bears and black bears did not differ in terms of habitat attributes at either spatial scale (*P*≥0.2; Table 2). Thus, distributions of sun and black bears were not partitioned according to the habitat attributes we measured.

**Table 2:** Habitat attributes at sites where fresh signs (<3 months) of sun bears and black bears were detected in Thung Yai Naresuan Wildlife Sanctuary, Thailand, 2001–2003.

Habitat	Spatial scale	Attribute	Sun bear		Black bear		*P*	Overall habitat	
			Mean	SD	Mean	SD		Mean	SD
MDF	Landscape	SEF in 6 km^2^ buffer (%)	43.8	19.1	44.3	27.7	0.61	38.5	24.2
		SEF in 21 km^2^ buffer (%)	55.3	11.2	51.4	21.6	0.78	49.4	21
		SEF in 30 km^2^ buffer (%)	58.2	10.8	54.3	19.5	0.82	52.2	20
		SEF in 150 km^2^ buffer (%)	59.7	8.5	56.6	10.3	0.36	55.8	11.2
		Distance to edge (m)	353	208	508	589	1	554	619
		Distance to water (m)	484	327	490	341	0.91	487	281
		Distance to disturbance (m)	3928	3829	2754	3513	0.82	3318	3551
	Local	Fruiting tree density (trees/ha)	7.1	2.9	6.1	3.5	0.32	4.8	3.7
		Fruit abundance index	7.9	5.5	7.3	5.2	0.79	5.9	5
		Canopy height (m)	19.9	4.3	18.5	4.7	0.49	20	4.6
		Canopy cover (%)	36.5	10.8	34.1	14.9	0.87	34	12.5
		Ground cover (1 to 5)	3.6	1.4	3.5	1.5	0.95	3.6	1.4
		Elevation (m)	814	74	799	82	0.96	792	73
SEF	Landscape	SEF in 6 km^2^ buffer (%)	79.8	19.7	76.4	20.5	0.61	76.4	21.1
		SEF in 21 km^2^ buffer (%)	71	17.8	68.9	18.8	0.72	71.1	17.3
		SEF in 30 km^2^ buffer (%)	68.9	16.9	67	17.9	0.66	69.4	16.8
		SEF in 150 km^2^ buffer (%)	60.4	14.4	57.4	15.4	0.64	61	14.5
		Distance to edge (m)	783	755	724	699	1	765	717
		Distance to water (m)	512	391	579	321	0.66	518	371
		Distance to disturbance (m)	3885	3170	3470	2763	0.92	3788	3209
	Local	Fruiting tree density (trees/ha)	8.3	5.1	7.4	4.3	0.61	6.9	5.5
		Fruit abundance index	4.5	3.1	4.3	2.8	0.88	3.9	3.4
		Canopy height (m)	27.7	5.2	26.7	5	0.53	27.5	4.4
		Canopy cover (%)	63.8	13.8	61.7	14	0.61	63.3	13
		Ground cover (1 to 5)	3.1	1.2	3.2	1.3	0.8	3	1.2
		Elevation (m)	822	72	819	79	0.45	817	69
MEF	Landscape	MEF/SEF in 6 km^2^ buffer (%)	100	0	100	0	1	100	0
		MEF/SEF in 21 km^2^ buffer (%)	100	0	100	0	1	100	0
		MEF/SEF in 30 km^2^ buffer (%)	100	0	100	0	1	100	0
		MEF/SEF in 150 km^2^ buffer (%)	98.9	0.14	98.9	0.12	0.74	98.9	0.1
		Distance to edge (m)	3193	161	3167	121	0.76	3191	184
		Distance to water (m)	891	270	826	222	0.36	842	207
		Distance to disturbance (m)	10611	865	10853	320	0.76	10453	489
	Local	Fruiting tree density (trees/ha)	13.3	9.4	18.9	5.1	0.37	13.9	9
		Fruit abundance index	7.5	7.8	11	5.3	0.37	7.3	6
		Canopy height (m)	20	0	17.1	2.6	0.2	17.7	2.6
		Canopy cover (%)	68.8	8.8	66.7	7.2	0.74	64.6	16.6
		Ground cover (1 to 5)	1.5	0	1.5	0.5	1	1.3	0.4
		Elevation (m)	1560	28	1607	58	0.36	1593	41.3

*P*-values are from Mann-Whitney tests of differences between bear species. Overall habitat values are means from all transects in that habitat. Local scale attributes reflect conditions immediately around transects; landscape scale attributes reflect the surrounding environment in home-range sized circles around transects. No significant differences between species were detected in any habitat. MDF: mixed deciduous forest; SEF: semi-evergreen forest; MEF: montane evergreen forest; SEF/MEF: combined evergreen forest.

### Habitat selection models

Fruiting tree density was included in all models with considerable support (ΔAIC <2) for both sun and black bears (Table 3). Elevation, percent evergreen forest, and fruit abundance index had tolerances <0.2 indicating problematic multicollinearity. We removed elevation, as it was correlated with canopy cover and fruit density, which are biologically more direct predictors of bear use. Fruit abundance index was correlated strongly with fruiting tree density; we retained the latter since it was less subjective and reflected similar information (food availability). Percent of evergreen forest in small and large circular buffers were correlated for each bear species; the larger was retained.

**Table 3:** Comparison of logistic regression models of habitat attributes influencing occurrence of black bears and sun bears in Thung Yai Naresuan Wildlife Sanctuary, Thailand, 2001–2003.

Bear species, Spatial scale	Model parameters	*X* ^2^	*P*	–2LL	AIC	ΔAIC	*w*_i_
*BLACK BEAR*							
Combined	Fruit, Canopy ht, Dist. to edge, Forest type	11.11	0.02	85.61	95.61	0.00	0.35
Combined	Fruit, Canopy ht, Dist. to edge	8.06	0.04	88.66	96.66	1.05	0.21
Combined	Fruit, Canopy ht, Dist. to edge, Forest type, Sun bear activity	11.17	0.05	85.55	97.55	1.94	0.13
Local	Fruit, Canopy ht	4.90	0.08	91.82	97.82	2.21	0.12
Local	Fruit	2.41	0.12	94.31	98.31	2.70	0.09
Local	Canopy ht	1.81	0.17	94.83	98.83	3.22	0.07
Local	Fruit, Canopy ht, Canopy cover, Ground cover, Sun bear activity	5.60	0.35	91.11	101.11	5.50	0.02
Landscape	% SEF, Dist. to edge, Dist. to water, Dist. to disturbance	1.16	0.89	95.56	105.56	9.95	0.00
Combined	All variables	9.32	0.41	87.40	107.40	11.79	0.00
*SUN BEAR*							
Local	Fruit	2.92	0.08	87.17	91.17	0.00	0.53
Local	Fruit, Black bear activity	3.21	0.20	86.88	92.88	1.71	0.23
Combined	Fruit, Dist. to edge	2.97	0.23	87.12	93.12	1.95	0.20
Local	Fruit, Canopy height, Canopy cover, Ground cover, Black bear activity, Black bear activity × Ground cover	3.87	0.69	86.22	98.22	7.05	0.02
Landscape	% SEF, Dist. to edge, Dist. to water, Dist. to disturbance	1.59	0.81	88.50	98.50	7.33	0.01
Local	Fruit, Forest type	1.25	0.54	97.05	103.05	11.88	0.00
Combined	All variables	3.23	0.98	95.07	115.07	23.90	0.00

Local scale variables reflect conditions immediately around bear signs; landscape scale variables reflect surrounding environment in home-range sized circles around bear signs. Fruit refers to density of fruiting trees. –2LL: –2 log likelihood. AIC: Akaike's Information Criterion. ΔAIC: Change in AIC. *w*_i_: model weight.

Standardized residuals of all models for each bear were between −1.6 and 1.3, indicating no points for which models fit poorly [34]. No models deviated from a logistic fit (Hosmer-Lemeshow tests: *P*>0.12). Cook's distance values were mostly very low (median 0.03–0.04), indicating few points with undue influence. However, in sun bear models Cook's distance was 3–6 times higher for MEF transects than all others, indicating undue influence on sun bear regression models [34]. Therefore, we constructed regressions for sun bear omitting MEF transects.

Both bear species tended to select habitats with higher and less variable fruiting tree density: transects with fresh signs of black and sun bears, respectively, had mean 8.1±5.4 and 8.2±4.8 fruiting trees/ha, whereas transects without fresh signs had 5.7±5.8 and 5.6±6.1 (*U* = 448.5, *P* = 0.05 for black bears; *U* = 402, *P* = 0.01 for sun bears); and median fruiting tree density was over two times higher in transects with (black and sun bears, respectively: 7.5, 6.7 fruiting trees/ha), than without (3.3, 3.3) fresh signs. Black bears also selected habitats with lower canopy heights (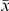 *= *18.5±4.7 m vs. 21.1±4.3 m for transects with vs. without fresh signs) that were closer to habitat patch edges (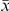 = 833±935 m vs. 930±987 m for transects with vs. without fresh signs) (Table 4). Additionally, they appeared to select evergreen over deciduous forest, but the odds ratio CIs of this parameter were very wide and included 1 (i.e., statistically insignificant). Models with interspecific competition had some support, but weights of evidence were <0.5 times that of the best models without competition (Table 3). There was very little support for other local scale variables related to forest structure (canopy cover, ground cover), or for landscape-level variables related to disturbance, habitat composition, or water. Classification success (with cutpoint  = 0.5) for top models was 65% for both black bears and for sun bears; with no covariates, classification rates were 57% and 51%. Nagelkerke's *r* ^2^ values were 0.20 and 0.06 for black bear and sun bear top models, respectively.

**Table 4:** Parameter estimates of best-fit models describing habitat selection by black bears and sun bears in Thung Yai Naresuan Wildlife Sanctuary, Thailand, 2001–2003.

Bear species	Parameters	*B*	SE	*P*	Odds ratio	95 CIs
Black bear	Fruit	0.12	0.05	0.03	1.13	1.01–1.25
	Canopy height	−0.17	0.07	0.01	0.84	0.73–0.96
	Distance to edge	−0.001	<0.0001	0.03	0.99	0.99–1.00
	Forest type	−1.31	0.78	0.09	0.27	0.06–1.25
	Constant	4.25	1.97	0.03		
Sun bear	Fruit	0.09	0.05	0.09	1.09	0.99–1.21
	Constant	−0.55	0.40	0.17		

Fruit refers to density of fruiting trees. Forest type is the proportion of deciduous forest (MDF).

### Interspecific relationships

Within-year signs of black bears and sun bears were found in 50 (70%) and 57 (80%) transects, respectively; fresh signs of each species were found in 30 (42%) and 34 (48%) transects, respectively. Sixty-two percent of transects had within-year signs of both species, and 21% had co-occurring fresh signs, similar to what would be expected by chance (product of the percent of transects with signs of each individual species: 70%×80% = 56% for within-year signs; 42%×48% = 20% for fresh signs). Accordingly, logistic regression models for each species that included fresh foraging activity of the other species received substantially less support than models without competition (Table 3). Likewise, co-occurrence of sun and black bears was not significantly different from random, for either fresh signs (*C*-scores: observed  = 285, expected  = 312.1; *P* = 0.46) or within-year signs (*C*-scores: observed  = 126, expected  = 144.3; *P* = 0.43). Though occurrence of each species was independent of the other, the extent of fresh foraging (signs/ha) by each species was negatively correlated (*n* = 49, *r* = −0.28, *P* = 0.05).

## Discussion

### Habitat use

Our observations of bear sign indicated that both sun bears and black bears regularly climbed trees in each of the three main habitats of Thung Yai. Evidence of feeding (broken branches, fresh climbing on trees with fruit, both fresh and year-old marks on the same trees) occurred on 70% of freshly-climbed trees (*n* = 86/123), indicating that bears climbed mostly to feed on fruits [27]. Other trees may have been climbed for other reasons, particularly shelter (i.e., rest, escape). Signs of insectivory were much less common and insects appeared in a correspondingly low proportion of scats. The paucity of insect-feeding sign was unlikely to be an artifact of low detection because such sign is conspicuous, and where bears feed mostly on insects, insect-feeding sign is much more prevalent relative to climbed trees [16], [39]. Insects were a relatively high proportion of the sign sample only in MDF (Table 1), perhaps because of the lower fruiting tree density there (Table 2) and correspondingly lower rate of tree climbing (Fig. 3). The higher density of opened logs in SEF may reflect higher tree density and thus more logs on the ground; also, periodic burning likely reduces availability of insect-laden logs in MDF (pers. obs.).

Foraging activity of both species was concentrated in evergreen forest types, probably because evergreen forests have higher density and species richness of fruit-bearing trees than MDF [25], [27]. Bears often concentrate their use of the landscape where food production is highest [40], [41]. Bear sign density should reflect relative time spent by individuals and(or) number of individuals, so the significant species-specific differences for within-year signs (Fig. 3) suggest that black bears most frequently used MEF and sun bears SEF. Although we sampled MEF in just one season, both fresh and within-year signs of black bear were consistently more abundant than sun bear (Fig. 3); as older signs reflect habitat use into the recent past (about 1 year), this indicates that black bears were the more active or abundant species in MEF throughout the year, not just in the season we surveyed.

Our results indicate that habitat use and behavior of these two species of bears in Thung Yai were strongly influenced by availability of food, as shown for other species of bears [42]. Of the 10 variables that we examined, including interspecific activity, fruiting tree density was most prominently related to presence of signs. With each additional fruiting tree per ha, the odds of encountering fresh signs increased 9% for sun bears and 13% for black bears. Black bear signs also tended to occur in transects with lower canopy height, perhaps indicating that larger-bodied bears tended to climb shorter trees. Whereas the correspondence between feeding signs and food availability is nearly tautological, others similarly observed, using radiotelemetry, that activity of American black bears was consistently concentrated in habitats with high fruit abundance [43], and site selection by American black bears [44] and grizzly bears [45] corresponded strongly to the availability of food.

The best models for each bear species explained modest amounts of variation in sign occurrence, suggesting that bears selected additional attributes that we did not measure, or that our measurements were coarse. Another explanation is that bears ate green vegetation or fallen fruits from the ground, resulting in habitat use that we could not detect. If one of the species ate more fallen fruits than the other, that could reduce competition despite shared preferences for the same foods; however, this probably would not account for much unexplained variation in the model as the fruit, whether on trees or fallen, would be at the same sites. The relatively poorer performance of the sun bear habitat model probably stemmed from our failure to incorporate sufficient predictors related to insect foods.

Overall classification success of logistic regression models is determined by the sum of absences and presences that are correctly predicted [46]. Although overall classification success of our models was only 65%, models had relatively low false positive error rates (29% and 33% of transects for black and sun bears, respectively). Thus, we considered models reasonably robust for our purposes, as we were interested mainly in identifying and assessing potentially important biotic and abiotic influences on bear species distribution, so sought to minimize spurious relationships (i.e., false positives).

### Interspecific relationships

Signs of both bear species occurred in all three forest types, so there was no evidence of strict habitat partitioning. Habitat partitioning appeared most evident in MEF, where black bear activity peaked at 14 signs/ha, and sun bears were much less common despite abundant fruit (Fig. 3, Table 2). However, the substantial overlap between sun bears and black bears in MDF and SEF, which comprise most (85%) of Thung Yai's forest cover and thereby constitute the main living space for both species, suggests that these two forest types contribute strongly toward support of both species.

The two bear species exhibited extensive spatial overlap: within-year signs of each co-occurred on >60% of transects. However, fresh foraging activity (i.e. signs/ha) for the two species was inversely related, indicating that although both species used the same foraging sites, they may not have been there at the same time. We offer two different interpretations of this finding. One is that this is evidence of competition on a fine spatial scale. Sun bears may have avoided feeding in food patches where black bears had recently been feeding in order to stay away from potential confrontations (interference competition); smaller carnivore species typically avoid larger ones [47]. Conversely, the larger-sized black bears, which maintain high intake rates by focusing on patches with high fruit density [14], [48], might avoid patches already harvested by sun bears because of diminished fruit density (exploitation competition). The fact that both species fed in patches with high fruit density (Table 2) implies that such patches were abundant in the forest, and that interspecific competition did not cause one or the other to be displaced to poorer feeding sites. An alternative explanation, which does not involve competition, stems from the solitary nature of bears: fresh signs on a transect may tend to be from only one species because they resulted from the activities of only one bear. If two or more bears of the same species rarely fed in the same transect within the same 3-month time (perhaps because foods were well dispersed on the landscape), then the same would be true for two different species that shared these same foods.

Other studies that used more direct research methods have revealed instances of fine-scale spatial avoidance by coexisting competitors that share similar diets. For example, radio-tracked spotted-tailed quolls (*Dasyurus maculatus*) and two other competing carnivore species occupied small areas (about 1–2 km^2^) simultaneously within overlapping home ranges without direct conflict [49], implying fine-scale avoidance. Similarly, despite high densities and small, overlapping home ranges, bobcats (*Lynx rufus*) coexisted with coyotes (*Canis latrans*) through spatial avoidance, probably using scent [50]. Likewise, mule deer (*Odocoileus hemionus*) and elk (*Cervus elaphus*) strongly avoided each other over short time periods (6 hours), but this effect dissipated when viewed over a longer period of 7 days [51]; this finding corresponds to our interpretation that bears avoided each other at short time scales but their habitat use was highly similar over longer time periods.

### Shared preferences and coexistence

The patterns of habitat selection by sun bears and black bears that we observed were consistent with a model of the niche based on shared preferences. First, both species co-occurred extensively throughout the main forest types of SEF and MDF, they ranked forest types similarly (evergreen > deciduous), and they keyed on the same ecological attribute (fruit abundance). Though this characterization pertains just to their realized niches, the fact that their distributions in Thung Yai were not constrained by presence of the other species, and that elsewhere in Southeast Asia sun bears are relatively common in montane forest in the absence of black bears, indicates that their fundamental niches (i.e., the niche in the absence of competitors) overlap significantly. Second, at the same time, their habitat use differed in ways that would be expected based on their physical differences, with larger black bears apparently dominating the most fruit-rich, and presumably most preferred habitat (MEF), and smaller sun bears more apt to feed on less-preferred insects. These features―overlapping fundamental niches combined with asymmetric competition―are salient features of the shared preference niche [3], [4]. Communities structured through shared preferences, in which species' habitat use overlaps widely but the larger species is predominant where resources are most abundant, occur among diverse taxa, including salmon [52], hummingbirds [53], rodents [7], and shrews [54].

Limited sampling effort in MEF raises the possibility that the preponderance of black bear sign there was a sampling artifact. Combining MEF with SEF transects into a general evergreen forest category yields mean sign densities that are even more equivalent between the two species (sun and black bear, respectively, mean fresh signs/ha: 2.5±3.6, 1.9±2.8, *U* = 900.5, *P* = 0.53; within-year signs/ha: 8.3±7.3, 6.9±6.3, *U* = 837, *P* = 0.27) than in SEF alone (Fig. 3). That is, if the apparent competitive exclusion of sun bears in MEF was merely a sampling artifact, the use of habitats by these two species is even more shared, and the main observed difference between them is in their use of insects. While more research is warranted to clarify whether black bears truly limit sun bear use of MEF, we suggest that such exclusion fits with their behavior: in food-rich sites in other parts of their range, adult male Asiatic black bears even exclude subadult males and females of their own species [31], [55]. Our inference that black bears were predominant in MEF also corresponds with recent camera trap data from 24 other sites in Southeast Asia where these two species are sympatric, and where black bears tended to be the more commonly photographed species at higher elevations [56]. Distinct use of some resources by black bears and sun bears might provide a refuge from competitive effects, enabling each to maintain a sufficiently high density, and hence coexistence, despite extensive habitat overlap at low elevations and shared preferences for most resources [57].

Bear coexistence in Thung Yai appears structured chiefly though shared preferences, but mixtures of preferences can also occur in nature [4]. In the case of these two species of bears, the one-sided use of insects by sun bears might either signify exploitation of a less-preferred food source in response to present competition with black bears for more-preferred fruits (the shared preference paradigm) or may reflect differences in morphology of the two species of bears (perhaps stemming from competition on an evolutionary time scale). Sun bears have (for their size) unusually large canines and robust jaw musculature [58], [59], which seem especially suited for breaking into protected insect nests, and their small body size probably enhances their ability to cling to a tree trunk while doing so (e.g., excavating *Trigona* nests from live hardwood trees). These physical differences may promote distinct (divergent) preferences that lead to resource partitioning, although we emphasize that insects are a minor part of the diets of both species in Thung Yai, so adaptations for insectivory are likely to be less important in shaping their coexistence than their shared preferences for fruit.

Why might black bears have predominated in MEF? Fagaceae (oaks) and Lauraceae (cinnamon)—fruit tree families preferentially used by both sun and black bears in Thung Yai—are exceptionally abundant in this habitat (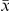 = 138 trees >10 cm DBH/ha, 27% of stem density) compared with SEF and MDF (61 trees/ha, 11% of stem density) [25], [60]. Interference competition would be intensified where preferred foods are spatially concentrated. In North America, for example, larger brown bears often exclude American black bears from dense, defensible patches of food like salmon streams, berry patches, and cutworm moth (*Euxoa auxiliaris*) aggregation sites [19], [61]–[63]. Likewise, the high density of preferred fruit trees in montane forest in Thung Yai probably attracts relatively high densities of black bears, making sun bears less apt to use this habitat. Black bears climbed 7% of available Fagaceae and Lauraceae in MEF, compared with just 3% of these families in SEF [27], suggesting higher black bear density in MEF. Competitive coexistence is more likely when food density is low or intermediate [64], which probably explains the greater overlap by sun and black bears in SEF and MDF compared to MEF. The MEF site also had much sparser ground cover than SEF and MDF (Fig. 2, Table 2), which may be another factor that dissuaded sun bears from using it. Subordinate species often avoid open areas in favor of sites with denser cover that are safer [46], [47], even if the denser sites have less food [65].

The overall scarcity of sun bear signs relative to black bear in higher-elevation forest in Thung Yai also may have been related to the paucity of insects there. Biomass and richness of ants and termites declines sharply with increasing elevation in the tropics [66], [67]. We observed no termite mounds or bee nests in montane forest at our site. Though both bear species consume insects, sun bears are often described as more insectivorous [13]. Insects are the predominant food items for sun bears in Indonesia and Malaysia, except during mast fruiting events when they become almost completely frugivorous [15], [16], [39]. In contrast, insects constitute a small proportion of diets of Asiatic black bears (0–4% relative volume) throughout their range [14], [30], [68]–[70]. Sun bears, because of their smaller size and presumably lower absolute food requirements, may be better able than black bears to subsist on scattered insects.

In communities structured by shared preferences, subordinate competitors (typically the smaller species in the case of interference competition) should expand their niche after removal of the dominant species, whereas removal of the subordinate species should produce little change in resource use by the dominant species [4]. Although testing this experimentally for bears would be impossible, patterns of habitat and resource use by sun bears and black bears in parts of their respective ranges where the other species is absent conform to these predicted responses. In temperate Asia, where sun bears are absent, black bears rely mostly on fruit and vegetation, and insects comprise a minor portion of their diet, as in Thung Yai (citations above). In Sundaic Southeast Asia, where black bears are absent, sun bears occur up to at least 2000 m [71]; they were frequently camera-trapped (100 photos in 4536 trap nights) between 700–1940 m in Sumatra [72]. Thus, it appears that sun bears might use the montane forest in Thung Yai more if it was not occupied by a high density of a larger competitor. This situation is perhaps intensified because insects, which are an important supplementary food for sun bears, are largely unavailable, and ground cover is sparse.

Empirical tests of predictions from coexistence theories, such as conducted in this study, are important for understanding mechanisms that maintain diversity [73]. Our results imply that conservation of co-occurring sun bears and black bears requires protection of fruit-rich habitats, as well as maintenance of alternative foods (i.e., insects) and habitat heterogeneity (which presents a combination of high and low fruit densities), as these conditions might facilitate ecological partitioning that underlies their coexistence. A distinct possibility is that these two species presently share most resources mainly because both species are well below carrying capacity due to hunting [74], so resources are non-limiting. The situation could become quite different if better protection from human-caused mortality leads to increased bear populations and thus more interspecific competition.

### Limitations

Our indirect sampling approach incurred three important limitations for the questions we addressed. First, both species of bears not only climb to obtain fruit, but also eat fallen fruit from the ground [14], [16]. Because foraging on fallen fruit leaves little or no trace, a portion of feeding events were missed in our study transects. Our interspecific comparisons implicitly assumed that such terrestrial fruit feeding was similar for the two species. Second, the method that we used to distinguish black and sun bear claw marks, though accurate for adult animals, often fails to distinguish black bear cubs from sun bears [32]. Thus, the sun bear portion of the sample may have been slightly inflated (if some were really black bear cubs that climbed independent of their mother). Accounting for this would indicate that the use of SEF and MDF was even more equitable for the two species, and use of MEF even more exclusive to black bears (Fig. 3). Our main conclusions—that habitat use by each species overlapped substantially in SEF and MDF but diverged strongly in MEF—are thus actually stronger than indicated by our analyses. Third, we could not assess whether temporal partitioning contributed to the coexistence of bears. Diel partitioning of activity can facilitate coexistence between competitors [75]. Bears in Thung Yai may alter their activity in response to the other species, as has been shown for other sympatric bears [20]. We doubt, though, that temporal partitioning is fundamental to the coexistence of these two species, both of which have been observed or camera-trapped in Thung Yai diurnally and nocturnally (W. Chutipong, unpublished data).
